# Bruising, hives, and joint pain in a 3-year-old boy

**DOI:** 10.1016/j.jdcr.2024.11.015

**Published:** 2024-11-24

**Authors:** Disha Patel, Lauren Prusinski Fernung, Loretta S. Davis

**Affiliations:** a Department of Medicine, Medical College of Georgia at Augusta University, Augusta, Georgia; b Department of Dermatology, Medical College of Georgia at Augusta University, Augusta, Georgia

**Keywords:** arthralgia, drug reaction, ecchymoses, postinflammatory hyperpigmentation, serum sickness, serum-sickness like reaction, urticaria-like eruption

## History

**Fig 1:**
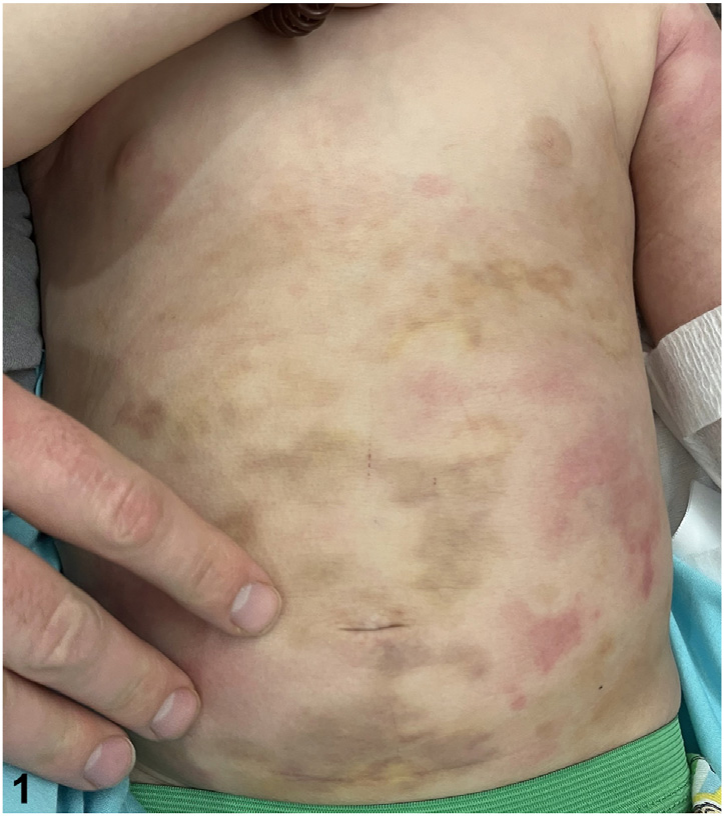

**Fig 2:**
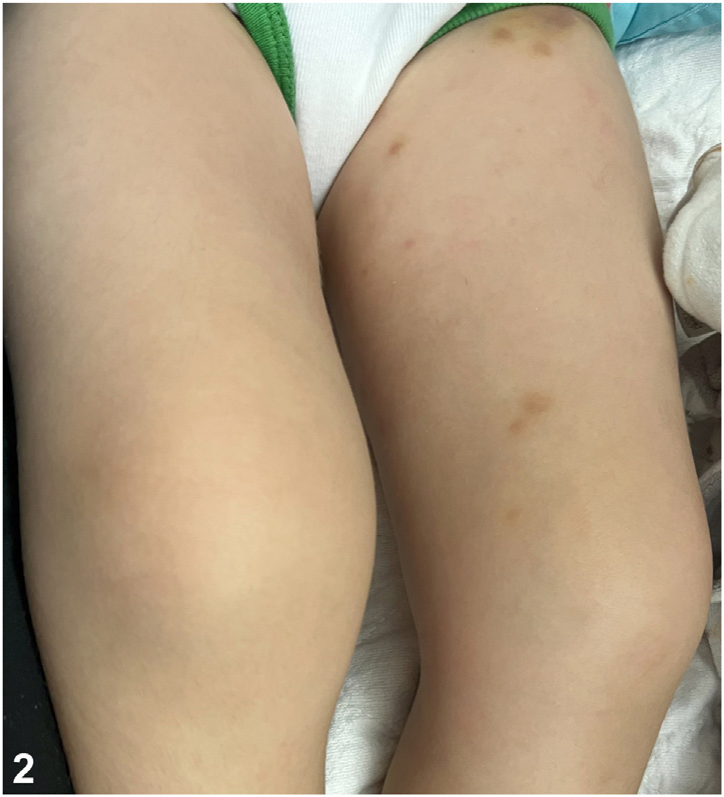

A 3-year-old boy presented to the emergency department for reported hives, bruising and joint pain 3 days after a 10-day course of amoxicillin for otitis media. Afebrile and playful, he could not bear weight due to pain. Pink-purple polycyclic, blanchable papules, patches, and thin edematous plaques and similarly shaped brown-yellow macules and patches were scattered on trunk, extremities, and groin without associated lymphadenopathy (Fig 1). Edematous wrists, knees, and ankles were tender to palpation (Fig 2). White blood cell count was 15 (4.5-11), erythrocyte sedimentation rate 23 (0-19), C-reactive protein 1.15 (0.00-0.50), and C3 complement 175 (80-150) with normal C4.

**Question 1: What is the MOST likely diagnosis?**

A. Child abuse
B. Serum sickness
C. Serum-sickness like reaction (SSLR)
D. Urticarial vasculitis (UV)
E. Urticaria multiforme

**Answers:**

A. Child abuse – Incorrect. Child abuse is unlikely given the child’s playful nature and symmetric, generalized arthralgias. Polymorphous shaped lesions are not typical of external trauma.
B. Serum sickness – Incorrect. Serum sickness is an immune-complex-mediated hypersensitivity reaction triggered by foreign protein; β-lactam antibiotics are not reported triggers. In addition to classic fever, polyarthritis, and diffuse rash, deposition of immune complexes causes multiorgan involvement and low levels of complement.^1^^,^^2^
C. Serum-sickness like reaction (SSLR) – Correct. SSLR is a hypersensitivity reaction which typically occurs in young children treated with antibiotics. Fever and arthralgias accompany blanchable urticaria-like papules and plaques which persist longer than 24-36 hours. Plaques are often annular or arcuate, becoming polymorphous in appearance as lesions coalesce. Pink plaques may become violaceous but remain blanchable and are not reticulated as seen with vascular injury. The overall reaction lasts days to weeks following withdrawal of the offending medication. Resolving lesions classically develop an ecchymotic-appearance and heal with hyperpigmentation. Erythrocyte sedimentation rate and C-reactive protein are frequently elevated.^1^^,^^3^^,^^4^
D. Urticarial vasculitis (UV) – Incorrect. UV is a rare form of leukocytoclastic vasculitis, typically seen in adults. Urticaria-like lesions last >24 hours and heal with ecchymotic hyperpigmentation. The process is typically chronic with recurrent episodes. Biopsy is required for diagnosis. Hypocomplementemic and normocomplementemic variants exist. The scenario of a single acute episode in a young child argues against UV as the best answer.^3^^,^^5^
E. Urticaria multiforme – Incorrect. Urticaria multiforme is a unique variant of acute urticaria which typically affects young children. Annular and polycyclic wheals with dusky centers last less than 24 hours and heal without hyperpigmentation. Arthralgias are not typical.^1^^,^^3^

**Question 2: Which statement is TRUE for both SSLR and serum sickness?**

A. Arthralgia is a common symptom
B. Complement levels are typically decreased
C. Immune complex formation is present
D. Most common triggers are antibiotics
E. Multisystem involvement is typical

**Answers:**

A. Arthralgia is a common symptom – Correct. Arthralgia and edema of the feet and hands are commonly seen in both serum sickness and SSLR. Fever, urticarial-like rash, and pruritus are also typical of both conditions.^2^^,^^4^
B. Complement levels are typically decreased – Incorrect. In SSLR, complement levels are typically normal or slightly elevated as complement can be an acute phase reactant. In contrast, immune complex deposition in serum sickness activates the complement system resulting in complement consumption, commonly demonstrated as low C3 and C4.^2^^,^^4^
C. Immune complex formation is present – Incorrect. Serum sickness is the classic type III immune-complex-mediated hypersensitivity reaction which results in extravascular immune complex deposition. In contrast, SSLR lacks the formation of immune complexes and is thought to occur via hypersensitivity to drug metabolites, although the pathogenesis is not entirely understood.^2^^,^^4^
D. Most common triggers are antibiotics – Incorrect. The most common offending agents for SSLR are antibiotics; historically, cefaclor and subsequently penicillins, cephalosporins, and sulfonamides have been reported. In one study of 83 patients, 82.7% of cases were attributed to amoxicillin as seen in this case. Other medications include bupropion, fluoxetine, and thiouracil. Several infections have also been linked to SSLR including streptococcus and hepatitis B. In contrast, serum sickness is caused by medication containing heterologous antigens such as vaccinations, immunomodulators, and antivenoms.^2^^,^^4^^,^^5^
E. Multisystem involvement is typical – Incorrect. Serum sickness is caused by extravascular immune complex deposition into parenchymal tissue and synovial joint fluid. The resultant inflammatory response may cause vasculitis, nephropathy, and neurologic complaints manifested as blurry vision, headache, and neuropathy. Lymphadenopathy has been reported but is not a hallmark of the disease. SSLR lacks immune complex formation; thus multisystem involvement is not expected.^2^^,^^5^

**Question 3: What is the MOST appropriate first-line treatment for this patient?**

A. Admission to the hospital for management
B. Immediate cessation of the offending medication and administration of plasmapheresis
C. Immediate cessation of the offending medication and short course of glucocorticoids
D. Immediate cessation of the offending medication and supportive care
E. Short course of glucocorticoids and administration of antihistamines

**Answers:**

A. Admission to the hospital for management – Incorrect. The patient is stable and supportive care can be managed in the outpatient setting. Indications for hospitalization include severe symptoms (high fever or severe arthritis) or uncontrolled infection for which the culprit drug had been prescribed.^2^^,^^4^
B. Immediate cessation of the offending medication and administration of plasmapheresis – Incorrect. While immediate cessation of the suspected triggering medication is correct, plasmapheresis is unnecessary. Plasmapheresis may be utilized in situations where it is not possible to immediately stop the offending medication.^2^^,^^4^
C. Immediate cessation of the offending medication and short course of glucocorticoids – Incorrect. High fever or severe arthritis and arthralgias may be managed with a short course of systemic glucocorticoids. This patient does not have these findings and is best managed conservatively with NSAIDs.^5^
D. Immediate cessation of the offending medication and supportive care – Correct. This is appropriate management for this patient whose symptoms are limited to the skin and joints and is otherwise stable. SSLR is a self-limited condition that typically resolves within 1 to 2 weeks after discontinuation of the offending medication. NSAIDs are recommended for treatment of mild arthralgia and fever. Pruritus is commonly managed with antihistamines.^1^^,^^5^
E. Short course of glucocorticoids and administration of antihistamines – Incorrect. High fever or severe arthritis and arthralgia, not present in this patient, may be managed with a short course of systemic glucocorticoids. Antihistamines may be included as supportive care. The most critical aspect of management is discontinuation of the offending agent.^4^^,^^5^

## Conflicts of interest

None disclosed.
